# Inhibition of the inflammatory cytokine tumor necrosis factor-alpha with etanercept provides protection against lethal H1N1 influenza infection in mice

**DOI:** 10.1186/cc13171

**Published:** 2013-12-27

**Authors:** Xunlong Shi, Wei Zhou, Hai Huang, Hongguang Zhu, Pei Zhou, Haiyan Zhu, Dianwen Ju

**Affiliations:** 1 Department of Biosynthesis, School of Pharmacy, Fudan University, 826 Zhangheng Road, Shanghai 201203, China; 2 Department of Chemistry, Fudan University, 220 Han Dan Road, Shanghai 200433, China; 3 Department of Pathology, Shanghai Medical College, Fudan University, 138 Yi Xue Yuan Road, Shanghai 200032, China

## Abstract

**Introduction:**

Factors implicated in influenza-mediated morbidity and mortality include robust cytokine production (cytokine storm), excessive inflammatory infiltrates, and virus-induced tissue destruction. Tumor necrosis factor-alpha (TNF-α) is an important pro-inflammatory cytokine present during influenza infection, but it is unclear whether direct inhibition of TNF-α can elicit protection against influenza infection.

**Methods:**

In this study, the commercially available TNF-α inhibitor etanercept was used to inhibit TNF-α induced by lethal A/FM/1/47 (H1N1) influenza virus infection of mice. The effects of TNF-α inhibition on mouse survival, pathologic changes, immune cell infiltration, inflammatory cytokine secretion, Toll-like receptor expression, and activation of the NF-κB (nuclear factor kappa B) signaling pathway were evaluated.

**Results:**

The intranasal delivery of etanercept provided significant protection against mortality (30% of mice survived up to 14 days after infection) in mice treated with etanercept. In contrast, no survivors were found beyond 6 days in mice treated with saline after lethal challenge with H1N1 influenza virus. It was observed that etanercept significantly reduced inflammatory cell infiltration (for example, macrophages and neutrophils), inflammatory cytokine secretion (for example, interleukin-6, TNF-α, and interferon gamma), and expression of Toll-like receptors (*TLR-3, TLR-4,* and *TLR-7*). Etanercept also downregulated and inhibited the cascade proteins of the NF-κB signaling pathway (for example, *MyD88, TRIF, NF-κB,* and *p65*), as well as enhanced host control of virus replication.

**Conclusions:**

These findings indicate that etanercept, by blocking TNF-α, can significantly downregulate excessive inflammatory immune responses and provide protection against lethal influenza infection, making its use a novel strategy for controlling severe influenza-induced viral pneumonia.

## Introduction

Influenza viruses are a major contributor to morbidity and mortality in humans, and with their ability to cause yearly epidemics and occasional pandemics, they represent a considerable burden to healthcare systems globally [1]. The recent emergence of the avian influenza A (H5N1) virus, the 2009 pandemic influenza A (H1N1) virus, and the novel avian influenza A H7N9 virus [2-4] served to highlight that respiratory viruses are important causative agents of severe pneumonia. The current battery of antivirals available in clinical practice for the treatment of viral pneumonia is limited [5]. Thus, the continued pandemic threat of these circulating viruses makes the identification and the development of novel therapeutic strategies an urgent matter.

Factors implicated in the high morbidity and mortality associated with influenza virus infection include, but are not limited to, a robust cytokine production (cytokine storm), excessive inflammatory infiltrates, virusnduced tissue destruction [6], and secondary bacterial coinfection [7]. Among these factors, the excessive cytokine response is considered to be the key contributor [8].

Corticosteroids and cyclooxygenase-2 (COX-2), which are both inhibitors of inflammation, have been tested as inhibitors of influenza virus-induced immunopathology, although their effectiveness was shown to be limited [9-11]. Other novel immunomodulatory drugs, such as sphingosine-1-phosphate receptors analogs, have been reported to provide protection against pathogenic influenza virus by suppressing the cytokine storm [12,13]. Therefore, effective inhibition of inflammation appears to be a promising therapeutic strategy for respiratory virus infections.

TNF-α, an important inflammatory cytokine, has been shown to correlate with morbidity and mortality in macaques [14] and humans [15,16] infected with highly virulent influenza viruses. However, the role of TNF-α in virus clearance and immunopathologic lung injuries during influenza virus infection is still controversial, and whether direct inhibition of TNF-α can elicit protection from influenza infection is still unknown.

Etanercept (brand name, Enbrel), an anti-TNF-α agent, is a fusion protein of the human p75 TNF-α receptor attached to the Fc portion of human IgG1 [17], which has been approved for the treatment of rheumatoid arthritis [18,19]. However, no evidence supports the protective effects of etanercept against influenza infection.

In this study, a murine model of lethal acute respiratory H1N1 influenza A infection and etanercept was used to investigate the immunoregulatory role of TNF-α in viral clearance, host immune responses, and lung immunopathology. For the first time, our study demonstrated that the inhibition of TNF-α had a significant effect on the extent of lung immunopathology and inhibited inflammatory cellular infiltration and cytokine responses. In addition, we observed a decrease in influenza virus replication and an increased survival of influenza virus-infected mice.

## Materials and methods

### Experimental infection of mice with a mouse-adapted H1N1 influenza virus

BALB/c male mice (16 to 18 g) were purchased from the Shanghai SLACCAS Laboratory Animal Co., Ltd. (Shanghai, China). Mice were housed under specific pathogen-free conditions and given free access to sterile water and standard mouse food. All experimental protocols were approved by the Animal Experiment Ethics Committee of Fudan University (Shanghai, China).

The influenza virus strain A/FM/1/47 (H1N1) used in this study is a highly virulent, mouse-adapted virus that was isolated from patients at Fort Monmouth, NJ, USA, during an outbreak in 1947. The virus can cause severe pneumonia and high mortality in mice (ATCC VR-97). The virus was supplied by the Shanghai Center for Disease Control and Prevention (Shanghai, China) and was stored in aliquots at −70°C. A freshly thawed aliquot was used for each experiment [20]. The TNF-α inhibitor etanercept was obtained from Beijing SL Pharmaceutical Co., Ltd. (Beijing, China).

Mice under isoflurane anesthesia were infected intranasally (i.n.) with 10 TCID_50_ of A/FM/1/47 (H1N1) [21]. Two hours after infection, mice were i.n. (local administration for respiratory system) inoculated with either 30 μl of 0.9% (wt/vol) NaCl (virus control) or 2.5 mg/kg of etanercept (dissolved in 0.9% (wt/vol) NaCl) twice daily. An uninfected control group of mice also received 0.9% (wt/vol) NaCl in the same manner.

### Mice survival and body-weight loss

For the survival study (*n* = 10 mice per group), etanercept or virus control was administered as described for 7 days. Then, mice were continuously monitored for survival and body-weight loss for an additional 7 days. These experiments were repeated 3 times, and we made related calculations.

### Histopathology

For histopathologic analysis, experimental infections as described earlier were performed. On day 4 after infection, mice (*n* = 6 per group) were euthanized and weighed. Lung tissues were harvested and weighed, and the corresponding lung/body index was calculated. The left lobes of the lung were immersed in PBS-buffered formalin, and were then preserved in paraffin blocks by using standard procedures. Tissue sections (10 μm) were cut, placed on glass slides, and stained with hematoxylin and eosin by using standard techniques.

A tissue-inflammation score was assigned to the analyzed sections of each lung by using the mean score obtained from six separate random fields per tissue section. Scores were assigned according to the percentage of lung pathologic congestive involvement, as follows: none, 0; ≤ 25%, 1; 26% to 50%, 2; 51% to 75%, 3; and ≥76%, 4.

Microscopic analysis was carried out by three separate pathologists who were blinded to the various experimental treatments.

### Inflammatory cytokine measurement

On days 2 and 4 after infection, mice (*n* = 6 per group) were euthanized, and lung tissues were harvested. These tissues were individually homogenized in PBS buffer, and the supernatants were used for inflammatory cytokine measurement. The quantities of TNF-α, IFN-γ, and IL-6 were determined by using commercial enzyme-linked immunosorbent assay (ELISA) kits (BD Biosciences) by following the manufacturer’s instructions. The average level of inflammatory cytokines was calculated according to these data from three separate experiments.

### Infiltrate immune cell analysis with flow cytometry

On days 2 and 4 after infection, mice (*n* = 4 per group) were euthanized. Lungs were harvested and diced by using surgical scissors. Diced tissue was suspended in 4 ml of DMEM containing 0.5 mg/ml collagenase from *Clostridium histolyticum* type IV (Sigma, USA), 50 U/ml DNase I (Sigma), and 1 mg/ml trypsin inhibitor type II-s (Sigma) for 1 hour at 37°C. The suspension was then crushed through a 40-μm basket filter, and unwanted red blood cells were lysed by using red blood cell lysis buffer containing 0.02 Tris–HCl (pH 7.4) and 0.14 NH_4_Cl. Inflammatory cells were purified by centrifugation in 35% (vol/vol) PBS-buffered Percoll (GE Healthcare Life Sciences, USA) at 500 *g* for 15 minutes. Cell pellets were resuspended in staining buffer (RPMI-1640 medium), and Fc receptors were blocked by using 25 μg/ml anti-mouse CD16/32. Cells were stained with fluorescently labeled antibodies against the following mouse proteins: CD11b^+^, F480^-^, Ly6G^+^ (neutrophils), CD11b^+^, F480^+^, and Ly6G^-^ (macrophage/monocytes), CD3e^-^, CD49b^+^ [natural killer (NK) cells], CD3e^+^, CD19^+^ (B cells), CD3e^+^, CD4^+^ (T-helper cells), CD3e^+^, and CD8a^+^ (cytotoxic T cells) [22,23]. All antibodies were purchased from BD Biosciences (USA). The average counting of immune cells was calculated from three separate experiments.

### Inflammatory signaling pathways (Toll-like receptors and NF-κB) and influenza virus replication

On days 2 and 4 after infection, mice (*n* = 6 per group) were euthanized, and lung tissues were harvested. These left-lung lobes were homogenized for RNA isolation. The isolation of total RNA and cDNA synthesis were performed by using the Trizol reagent (Invitrogen) and PrimeScript RT reagent Kit (DRR047A; Takara) according to the manufacturers’ recommendations. The primers for *TLR-3*, *TLR-4*, *TLR-7*, *MyD88*, *TRIF*, *NF-κB*, the influenza virus *M* gene*,* and glyceraldehyde 3-phosphate dehydrogenase (GAPDH) were as described in Additional file 1. By using cDNAs as templates, quantitative real-time PCR was carried out by using the SYBR Green PCR Master Mix (Applied Biosystems) in a StepOne Plus Real-Time PCR Detection System (Applied Biosystems), according to the manufacturer’s instructions and with the following thermocycling parameters: 94°C for 5 minutes; followed by 94°C for 5 seconds, 60°C for 30 seconds for 40 cycles, with a final melting curve analysis of 60°C to 95°C. The mRNA expression levels were normalized to the corresponding expression level of the *GAPDH* housekeeping gene. The results of qPCR were from three separated independent experiments.

The remaining right-lung lobes were used for immunohistochemistry. Tissue sections (10 μm) were cut and processed as described earlier. The primary antibody, phospho-NF-κB p65 (Ser536) (93H1) rabbit monoclonal antibody (Cell Signaling Technology, Inc., USA) was used to evaluate the activation of the inflammatory NF-κB signaling pathway.

### Statistical analyses

All statistical analyses were performed by using GraphPad Prism for Windows (Version 6.0). The Gehan-Breslow-Wilcoxon test was used to analyze the survival of mice, whereas the one-way ANOVA was used to analyze other experimental data. In all cases, probability values less than 0.05 (*P* < 0.05) were taken to indicate statistical significance.

## Results

### Administration of the TNF-α inhibitor etanercept significantly improved survival of H1N1-infected mice and reduced pulmonary injury

**Figure 1:**
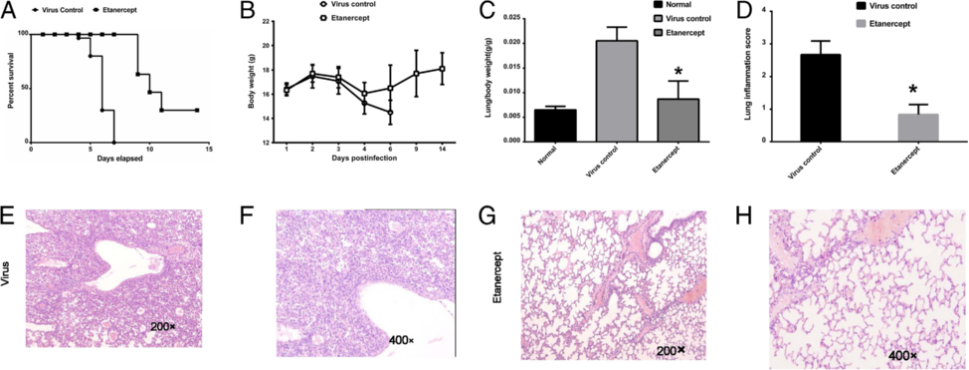
**Administration of etanercept protects against lethal H1N1 influenza virus infection and inhibits the immunopathologic effects associated with infection *in vivo.*** Saline or 2.5 mg/kg of etanercept was administered i.n. to mice 2 hours after i.n. infection with 10 TCID_50_ of influenza virus A/FM/1/47 (H1N1). **(A, B)** Mice were monitored for survival and body-weight loss. Data were derived from three separate experiments, with a total of 30 mice per group (10 mice per group each time). Survival curves show data until day 14 after infection, because further mortality was not observed at later time points. **(C, D)** Lung/body weight and inflammation score were evaluated on day 4 after infection. Mice were weighed (grams) and euthanized. Whole lungs were harvested, weighed (grams), and the corresponding lung/body index was calculated. The scores were calculated as follows: none, 0; ≤25%, 1; 26% to 50%, 2; 51% to 75%, 3; ≥76%, 4 lung involvement. #*P* ≤ 0.05 compared with saline recipients. **(E through H)** Histopathologic analysis on day 4 after infection was carried out by three separate pathologists who were blinded to the various experimental treatments.

Mice were treated with either saline or etanercept i.n. after intranasal infection with the lethal mouse-adapted human influenza virus A/FM/1/47 (H1N1). Etanercept administration significantly increased the survival of mice, compared with the control mice (Figure 1A). For recipients of saline alone, no mice survived beyond 7 days after infection, whereas 30% of mice that received etanercept survived until 14 days after infection, which was the end of the observation period (*P* < 0.01). In mice treated with etanercept, body-weight loss ceased on day 4 after infection and slowly recovered until the end of the experiment at day 14 (*P* < 0.05; Figure 1B). Obvious individual differences were also observed in etanercept-treated mice after 7 days, and whether they were related to ceasing of etanercept will be clarified in future work.

Lung/body index (Figure 1C) demonstrated that infection with H1N1 caused lung-tissue swelling and the production of significant amounts of exudate in the control mice. The administration of etanercept significantly alleviated lung swelling and exudate, an observation that was confirmed by the histopathologic analysis. Histopathologic analysis of lungs from the infected mice treated with etanercept revealed markedly reduced tissue injury, mononuclear cell accumulation, hemorrhage, and pulmonary edema (Figure 1E through H). In addition, etanercept significantly reduced tissue-inflammation scores compared with control mice on day 4 after infection (Figure 1D).

### Etanercept inhibited the burst of inflammatory cytokines and the recruitment of innate immune cells induced by lethal influenza virus infection

**Figure 2:**
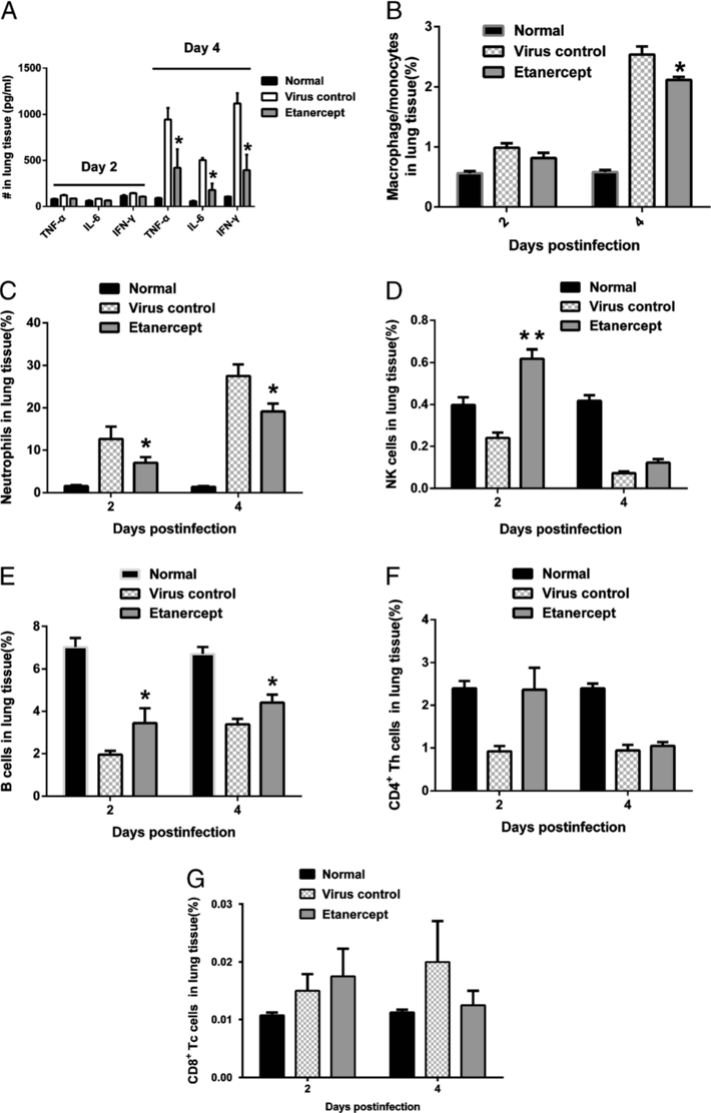
**Etanercept reduced inflammatory cells infiltration and dampened the production of pro-inflammatory cytokines.** On days 2 and 4 after infection, mice were euthanized, and the lungs were harvested to be homogenized individually and assayed for the following. **(A)** Inflammatory cytokines (TNF-α, IFN-γ, IL-6) were analyzed with ELISA. **(B through G)** Major immune cells (macrophage/monocytes, neutrophils, NK cells, B cells, CD4^+^ Th cells, and CD8^+^ Tc cells) were analyzed with flow cell cytometry. Four samples were picked up from mice per group each time for assaying, and three replicated experiments were conducted. Data are presented as mean ± SD. #*P* ≤ 0.05 compared with saline recipients.

Robust innate proinflammatory cytokine expression can cause direct tissue insult and recruit potentially tissue-destructive inflammatory cells. We selected three important inflammatory cytokines (TNF-α, IL-6, and IFN-γ) to evaluate the cytokine burst in lethally influenza-infected mice. ELISA results revealed that these cytokines increased in the virus-infected control mice as expected, but they were significantly inhibited in mice treated with etanercept (Figure 2A). The recruitment and infiltration of immune cells can be affected by inflammatory cytokine production. Analysis with flow cytometry revealed a consistent reduction in the accumulation of macrophage/monocytes (Figure 2B) and neutrophils (Figure 2C) in the lungs of mice treated with etanercept on 2 and 4 days after influenza A virus infection. Many significant differences were noted in neutrophils accumulation between virus control and etanercept-treated mice. The NK cell, an innate immune cell that plays an important role in controlling and clearing virus, count significantly decreased after infection. The administration of etanercept counteracted this reduction in NK cells (Figure 2D). At these same time points, the adaptive immune cells (CD4^+^ Th cells and CD8^+^ Tc cells) were also evaluated, but no clear influences of etanercept were observed in mice at days 2 and 4 after infection (Figure 2E through G). Even etanercept seems to alleviate the reduction of B cells after infection, but the possible influence of etanercept on adaptive immune responses must be explored in future work.

In agreement with the observations of diminished inflammation and pulmonary injury after influenza virus infection (Figure 1C through 1H), etanercept significantly inhibited inflammatory cytokine production as well as the accumulation of innate inflammatory infiltrates.

### Etanercept inhibited the activation of the NF-κB signaling pathway and enhanced host control of influenza virus replication

Before these experiments, we already used a pair of housekeeping genes *GAPDH* and *beta-actin* to assess these gene expressions under etanercept treatment, and we found the normalized results were consistent.

NF-κB (nuclear factor kappa B) family transcription factors are master regulators of immune and inflammatory processes in response to both injury and infection. Toll-like receptors (TLRs) recognize specific pathogen-associated molecular patterns (PAMPs) and can trigger the activation of the NF-κB pathway. In this study, we monitored the transcriptional levels of *TLR3/7* (specific for viruses), *TLR4,* and the downstream adaptor genes *MyD88* and *TRIF*, and *NF-κB p65*.

**Figure 3:**
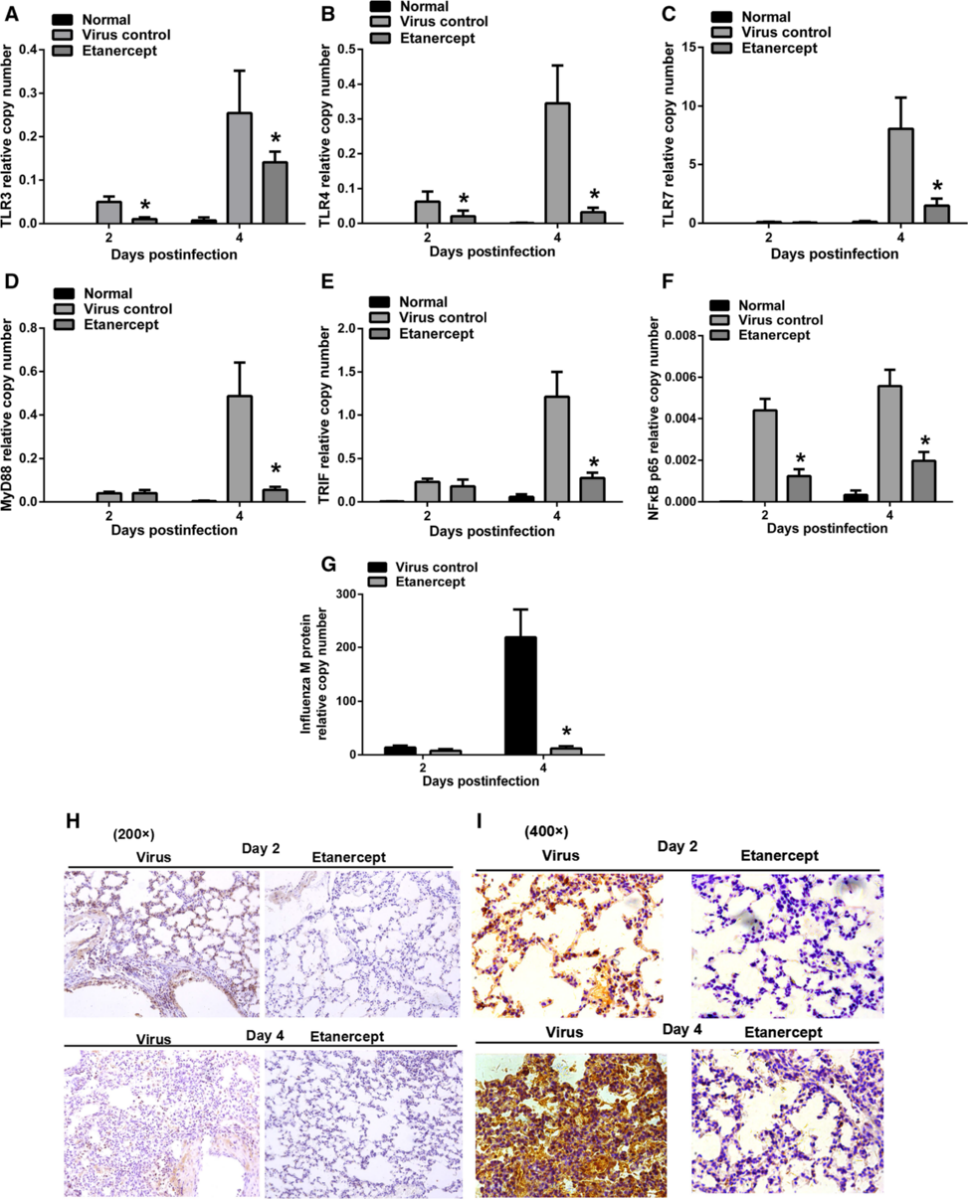
**Etanercept inhibited the TLRs-NF-κB signaling pathway and enhanced host control of influenza replication.** On days 2 and 4 after infection, mice were euthanized, and the lungs were harvested and assayed for the following: **(A through C)** Relative mRNA levels of *TLR3*, *TLR4,* and *TLR7*. **(D through F)** The cascade genes (*MyD88*, *TRIF*, *NF-κB, p65*) in the NF-κB pathway. **(G)** Influenza *M gene*, indicating the replication of influenza virus. **(H through I)** Immunohistochemical assay for phosphorylation of NF-κB p65 (dark yellow staining). The level of dark yellow staining indicates the activation of the NF-κB signaling pathway. Data are presented as mean ± SD. #*P* ≤ 0.05 compared with saline recipients.

Data from the qPCR revealed that administration of etanercept resulted in a reduced upregulation of the virus-specific *TLR3* and *TLR7* in mice induced by lethal influenza virus infection (Figure 3A and C), consistent with the decreased virus replication in mice treated with etanercept (Figure 3G). This indicated that the administration of etanercept enhanced the host control of influenza virus replication. Interestingly, the mRNA level of *TLR4*, the typical lipopolysaccharide (LPS) recognition receptor, was dramatically increased in virus-infected mice, which suggests that influenza virus infection resulting in complicated cross-activations, including the activation of *TLR4,* was also inhibited by etanercept (Figure 3B).

The overexpressed TLRs triggered the activation of the NF-κB signaling pathway, which was significantly influenced by etanercept administration, as seen by the downregulated mRNAs of *MyD88*, *TRIF,* and *NF-κB p65* in etanercept-treated mice (Figure 3D-F). The immunohistochemical results also clearly showed the phosphorylation of the NF-κB p65 protein, indicating the activation of the NF-κB signaling pathway in influenza virus-infected mice. In contrast, etanercept significantly reduced the phosphorylation of NF-κB p65 (Figure 3H through 3I).

These data strongly suggest that TNF-α may play an important role in the burst of inflammatory cytokines, recruitment of innate immune cells, and activation of the NF-κB signaling pathway. Blocking TNF-α resulted in improved survival and alleviated lung inflammation in influenza-infected mice.

## Discussion

TNF-α is traditionally considered a pro-inflammatory cytokine [24,25]; however, recent emerging evidence also identified an immune-regulatory role for it [26-28]. TNF-α blockers, such as infliximab, adalimumab, certolizumab pegol, golimumab, and etanercept, have been approved for the treatment of rheumatoid arthritis and other immune-mediated diseases. Thus, an interest exists in investigating the use of inhibitors of TNF-α in the treatment of infectious diseases [29,30].

In this study, we built up a simple and lethal influenza-infection mice model to evaluate the effects of TNF inhibitors in influenza virus-induced severe viral pneumonia. We administered etanercept to mice i.n. for a short period (7 days). The results showed that local inhibition of TNF-α could significantly reduce the high mortality in mice induced by lethal influenza infection, which indicated that TNF-α in lung tissue might play a pathologic role in severe virus infection. Even intranasal administration of etanercept (limited to the respiratory system) has been proved to be effective for lethal influenza infection in this research. Much remains to be investigated, such as patient screening and possible second bacterial infections.

Many cytokines/chemokines are essential for the control of virus replication but also exacerbate morbidity and tissue injury in mouse models [6]. IFN-γ activates inflammatory cells and stimulates the expression of multiple cytokines and chemokines [31-33]. IL-6 expression is directly linked to host morbidity [34,35], whereas TNF-α secretion enhanced pulmonary injury. In this study, a burst of IFN-γ, IL-6, and TNF-α was observed in lethal influenza virus-infected mice, especially on day 4 after infection. Blocking TNF-α obviously inhibited the overproduction of these inflammatory cytokines, which was consistent with the reduced lung injury and low mortality in mice treated with etanercept. The transcriptional and protein levels of other cytokines and chemokines still must be determined to assess the influence of etanercept.

Innate and adaptive immune cell infiltration contributes to lung inflammation. Our study showed that blocking TNF-α by using etanercept inhibited a large number of infiltrated neutrophils and macrophage/monocytes in influenza virus-infected mice. These results are similar to the findings on the functional roles of macrophages/neutrophils in virus replication and mortality [36]. Which kind of cell in lung tissue is targeted mainly by etanercept is under consideration in our future work. The influence on other immune cells such as NK, B, CD4^+^, and CD8^+^ cells on days 2 and 4 after infection was not so clear in mice treated with etanercept, and so more-detailed future studies will be performed at day 7 (and later) after infection to clarify this observation.

The NF-κB signaling pathway is a key factor controlling inflammatory cytokine secretion and inflammatory cell recruitment during virus infection. In this study, significant activation of the NF-κB signaling pathway was observed on days 2 or 4 after infection. This activation was inhibited in mice treated with etanercept. TNF-α can trigger the activation of the NF-κB signaling pathway via binding to tumor necrosis factor receptor (TNFR) [37], and so blocking TNF-α might inhibit the activation of NF-κB. Toll-like receptors may activate the signaling cascade in the NF-κB signaling pathway. The upregulation of *TLR4*, which recognizes oxidized phospholipids generated by reactive oxygen species (ROS) [38], was evidently inhibited in etanercept-treated mice. This indicated that blocking TNF-α might also alleviate influenza virus-induced inflammatory injury. But whether the reduction of *TLR4* is related to etanercept immunoadhesion is still unclear, and it will be explored in our future work. The inhibited virus-specific *TLR3/7* correlated with reduced influenza replication (*M* gene) in mice treated with etanercept, which indicated that blocking TNF-α enhanced host control of virus replication, but the elucidation of a possible mechanism requires more experimental investigation.

## Conclusions

In summary, blocking TNF-α by using etanercept suppressed the immunopathology and mortality in lethal influenza-infected mice. These effects may be ascribed to the inhibition of the cytokine bursts, reduced inflammatory cell infiltration, and downregulation of NF-κB signaling pathways.

This is the first attempt at etanercept use in influenza virus-induced viral pneumonia, and more details must be clarified, such as the influences of IFN system, adaptive immune responses, and different virus strains.

We envision that the use of etanercept (or other TNF antagonists), in combination with antiviral strategies, may be an effective tool against morbidity and mortality induced by seasonal and pandemic strains of influenza A virus.

## Key messages

• TNF-α inhibitor etanercept significantly improved survival and limited the lung inflammation in lethal influenza-infected mice.

• Blocking TNF-α markedly inhibited the burst of major inflammatory cytokines (for example, IL-6, IFN-γ, and TNF-α) in the lung tissue of influenza-infected mice.

• Blocking TNF-α reduced innate immune cell infiltration (macrophage/monocytes and neutrophils) in mouse lung tissue.

• Blocking TNF-α enhanced host control of influenza virus replication.

• Blocking TNF-α downregulated the mRNA of Toll-like receptors and inhibited the activation of NF-κB signaling pathways.

## Abbreviations

i.n.: Intranasally; IFN-γ: interferon gamma; IL-6: interleukin 6; NF-κB: nuclear factor kappa B; NK cell: natural killer cell; Tc cell: cytotoxic T cell; Th cell: T-helper cell; TLR: Toll-like receptor; TNF-α: tumor necrosis factor alpha.

## Competing interests

The authors declare that they have no competing interests.

## Authors’ contributions

XLS carried out all *in vitro* and *in vivo* experiments in this study and drafted the manuscript. WZ and HH carried out the immunoassays. HGZ performed the histopathology analysis. PZ participated in the design of the study and performed the statistical analysis. HYZ and DWJ conceived the study, participated in its design and coordination, and helped to draft the manuscript. All authors read and approved the final manuscript.

## Supplementary Material

Additional file 1: Table S1 The primers for quantitative real time PCR. These RT primers for *M* gene, *TLR3*, *TLR-4*, *TLR-7*, *MyD88*, *TRIF*, *NF-κB, p65,* and *GAPDH* analysis. Using cDNAs as the template, quantitative real-time PCR was carried out by using the SYBR Green PCR Master Mix (Applied Biosystems) in a StepOne Plus Real-Time PCR Detection System (Applied Biosystems), according to the manufacturer’s instructions. The mRNA expression levels were normalized to the corresponding expression level of the *GAPDH* housekeeping gene. Click here for file
