# Supplementary material for: Inhibition of the inflammatory cytokine tumor necrosis factor-alpha with etanercept provides protection against lethal H1N1 influenza infection in mice
Source: Crit Care. 2013 Dec 27;17(6):R301. doi: 10.1186/cc13171 (PMC4057515; doi:10.1186/cc13171)
Supplement: Additional file 1: Table S1 — The primers for quantitative real time PCR. These RT primers for M gene, TLR3, TLR-4, TLR-7, MyD88, TRIF, NF-κB, p65, and GAPDH analysis. Using cDNAs as the template, quantitative real-time PCR was carried out by using the SYBR Green PCR Master Mix (Applied Biosystems) in a StepOne Plus Real-Time PCR Detection System (Applied Biosystems), according to the manufacturer’s instructions. The mRNA expression levels were normalized to the corresponding expression level of the GAPDH housekeeping gene. [file cc13171-S1.pdf]

Table 1

Name	Oligo	Primer sequence	Predicted size(bP)	Genebank accession
influenza A virus M gene	Forward primer	5'-GACCGATCCTGTCACCTCTGAC-3'	106	JN391198(HIN1) JN391191(HIN1) JN391243(H1N2) 171~276
	Reverse primer	5'-AGGGCATTCTGGACAAAGCGTCTA-3'		
GAPDH	Forward primer	5'-ACCACCATGGAGAAGGCTGG-3'	528	XM_003820309 481~1008
	Reverse primer	5'-CTCAGTGTAGCCCAGGATGC-3'		
TLR3	Forward primer	5'-GCGTTGCGAAGTGAAGAA-3'	133	NM_126166
	Reverse primer	5'-TCAAGAGGAGGGCGAATA-3'		
TLR4	Forward primer	5'-GCACTGTTCTTCTCCTGCC-3'	294	NM_021297 61~354
	Reverse primer	5'-GTTTCCTGTCAGTATCAAG-3'		
TLR7	Forward primer	5'-GGTGGCAAAATTGGAAGATCC-3'	111	NM_133211 2927~3037
	Reverse primer	5'-AGCTGTATGCTCTGGGAAAGGTT-3'		
MyD88	Forward primer	5'-CCAGAGTGGAAAGCAGTGTC-3'	395	NM_010851
	Reverse primer	5'-GTCCTTCTTCATCGCCTTGT-3'		
TRIF	Forward primer	5'-CCACGTCCTACACGGAAGAT-3'	246	NM_019706 NM_207623 326~571
	Reverse primer	5'-AACAGCATCTGCAGCTACCA-3'		
NF-κB p65	Forward primer	5'-ATGTGCATCGGCAAGTGG-3'	294	NM_009045 1073~1366
	Reverse primer	5'-CAGAAGTTGAGTTTCGGGTAG-3'		
